# Navigating the Hepatic Immune Landscape With Fine Needle Aspiration of the Liver—An Emerging Technique

**DOI:** 10.1111/liv.70358

**Published:** 2025-10-21

**Authors:** Kate Diana Lynch, Fabiola Curion, Hing‐Yuen Yeung, Charlotte Rich‐Griffin, Devika Agarwal, Helen Ferry, Andrew Slater, Emma Louise Culver, Roger William Chapman, Satish Keshav, Paul Klenerman, Calliope Athena Dendrou

**Affiliations:** ^1^ Translational Gastroenterology and Liver Unit, NDM Experimental Medicine University of Oxford Oxford UK; ^2^ Faculty of Health and Medical Sciences University of Adelaide Adelaide South Australia Australia; ^3^ Department of Gastroenterology and Hepatology, Royal Adelaide Hospital Central Adelaide Local Health Network Adelaide South Australia Australia; ^4^ Nuffield Department of Medicine, Wellcome Centre for Human Genetics University of Oxford Oxford UK; ^5^ Institute of Computational Biology Helmholtz Center Munich Munich Germany; ^6^ Department of Mathematics, School of Computation, Information and Technology Technical University of Munich Munich Germany; ^7^ Nuffield Department of Orthopaedics, Rheumatology, and Musculoskeletal Sciences, Kennedy Institute of Rheumatology University of Oxford Oxford UK; ^8^ Department of Radiology, John Radcliffe Hospital Oxford University, Hospitals NHS Foundation Trust Oxford UK

**Keywords:** fine needle aspiration, flow cytometry, liver, primary sclerosing cholangitis, single‐cell, steatohepatitis

## Abstract

**Background and Aims:**

Hepatic immune cell analysis is critical for understanding chronic inflammatory liver diseases, such as primary sclerosing cholangitis (PSC) and steatotic liver disease. However, liver immunoprofiling is limited due to reliance on end‐stage disease liver explants. Fine needle aspiration (FNA) is a minimally invasive technique that can overcome these limitations. We evaluate the safety and efficacy of liver FNA to profile hepatic immune subsets in non‐infectious liver conditions.

**Methods:**

Flow cytometry and single‐cell RNA sequencing (scRNA‐seq) were used to compare the hepatic immune cell composition and gene expression to that of matched peripheral blood mononuclear cells (PBMCs).

**Results:**

We obtained liver FNAs from 38 patients. The median pain score was 0. No serious adverse effects were reported. Flow cytometry demonstrated enrichment of CD69^+^ T and natural killer (NK) cells in the liver (all *P*_*adj*_ < 0.05). ScRNA‐seq of 38 012 hepatic immune cells and 78 751 PBMCs in a patient subset showed specific enrichment of *CXCR6* ^+^ NK, CD8^+^ central memory T, and mucosal‐associated invariant T (MAIT) cells in the liver, and relatively lower CD4^+^ regulatory T cell (Treg) abundance (all *P*_*adj*_ < 0.05). Gene expression and cell–cell interaction analyses revealed increases in cytokine production, signalling, and responsiveness in hepatic immune cells compared to PBMCs.

**Conclusions:**

FNA sampling is a safe approach for investigating the inflammatory landscape of PSC and other liver diseases. Single‐cell profiling reveals that FNAs capture tissue‐specific immune cell types and gene expression differences, suggesting this sampling method may provide a basis for future experimental medicine analyses.

**Summary**

- Hepatic immune cell analysis is critical for understanding chronic inflammatory liver diseases, such as primary sclerosing cholangitis (PSC) and steatotic liver disease, particularly given the poor treatment options for these conditions.
- However, studying these cells has been complicated by the difficulty of sampling liver tissue. To overcome this challenge, we have evaluated fine needle aspiration (FNA) as a liver sampling technique.
- We find that it is safe, well‐tolerated, and enables the analysis of hepatic immune cells. Compared to matched blood immune cells from PSC and steatotic liver disease patients, the FNA‐derived hepatic immune cells are enriched for liver‐resident cell types and are more activated. FNA may therefore provide a basis for future studies of hepatic immune cells in disease to help develop new treatment strategies.

**Abbreviations**
AIH: autoimmune hepatitis
ALD: alcohol‐related liver disease
ALP: alkaline phosphatase
ALT: alanine transaminase
CCR9: CC‐chemokine receptor 9
CD: Crohn's disease
cDC: conventional dendritic cell
cMono: *CD14* ^+^ classical monocyte
DC: dendritic cell
DEG: differentially expressed gene
FC: fold change
FDR: false discovery rate
FNA: fine needle aspiration/aspirate
IBD: inflammatory bowel disease
IFN: interferon
INR: international normalised ratio
IQR: interquartile range
LIL: liver‐infiltrating lymphocyte
LSM: liver stiffness measurement
MAdCAM‐1: Mucosal Addressin Cellular Adhesion Molecule‐1
MAITs: mucosal‐associated invariant T cells
MASLD: metabolic dysfunction‐associated steatotic liver disease
MNP: mononuclear phagocyte
Mono: monocyte
n.d.: no data
n/a: not applicable
ncMono/Mac: *FCGR3A* ^+^ non‐classical monocyte/macrophage
NK: natural killer
PBC: primary biliary cholangitis
PBMCs: peripheral blood mononuclear cells
pDC: plasmacytoid dendritic cell
PSC: primary sclerosing cholangitis
scRNA‐seq: single‐cell RNA‐sequencing
TCM: central memory T cell
TEM: effector memory T cell
Th: CD4^+^ T helper cell
TNF: tumour necrosis factor
Treg: CD4^+^ regulatory T cell
UC: ulcerative colitis
UDCA: ursodeoxycholic acid
UMAP: uniform manifold approximation and projection
VCAM‐1: vascular cell adhesion molecule‐1

## 1 Introduction

The analysis of hepatic immune cells is vital to aid our understanding of chronic inflammatory liver diseases, such as primary sclerosing cholangitis (PSC) and metabolic dysfunction‐associated steatotic liver disease (MASLD). However, such analyses have historically been fraught with limitations such as the need for liver biopsies, which can be associated with serious complications, a reliance on liver explants and therefore a restriction to analysing only end‐stage liver disease, and technical challenges encountered when isolating leukocytes from liver tissue. The first description of leukocyte isolation from a liver biopsy for research was reported in 1977 [1], and subsequently, more refined techniques were developed to isolate mononuclear cells from liver explants, including the use of mechanical dissociation followed by density gradient centrifugation in 2010 [2].

More recently, fine needle aspiration (FNA) of the liver has emerged as a minimally invasive technique for obtaining reasonable numbers of hepatic mononuclear cells for experimental analysis [3, 4, 5]. It employs a much finer needle (22–25 gauge) as compared with that used in a liver biopsy (typically 16–18 gauge), and therefore has a lower rate of severe complications such as internal haemorrhage. Analysis of cells derived from liver FNA to date has been limited to mostly cytology and flow cytometry, and has been used predominantly in cancer and viral hepatitis [4, 5, 6, 7, 8, 9]. Application of more in‐depth, high‐resolution profiling methods, such as single‐cell RNA‐sequencing (scRNA‐seq), to the analysis of liver FNAs is currently confined only to studies of chronic hepatitis B [10, 11, 12].

However, the FNA approach is poised to lend itself well to the improved characterisation of the immunological landscape of non‐infectious inflammatory liver diseases such as PSC—particularly in the earlier stages of disease. This is critical given that PSC is a chronic biliary condition that leads to significant comorbidity and mortality; its underlying pathophysiology is still poorly understood, and consequently there is a paucity of therapeutic options beyond end‐stage liver transplantation, after which the disease often recurs [13, 14]. The combination of liver FNA with scRNA‐seq for the early‐stage profiling of chronic inflammatory liver diseases may help to elucidate disease‐relevant processes and to inform experimental medicine studies.

Here, we sought to assess the safety and efficacy of FNA of the liver as a technique to evaluate differing hepatic immune subsets in a range of liver conditions, including PSC and MASLD, using both flow cytometry and single‐cell transcriptomics, as well as comparing hepatic immune cell composition and gene expression to that of paired peripheral blood immune cells.

## 2 Materials and Methods

### 2.1 Ethics and Patient Information

All samples were obtained from patients with written informed consent under Research Ethics Committee approval (South Central—Oxford B Research Ethics Committee reference 17/SC/0290 and 09/H0603/19). The study protocol conformed to the ethical guidelines of the 1975 Declaration of Helsinki as reflected in a priori approval by the above‐mentioned human research committee. Details and protocols pertaining to patient sample acquisition, cell isolation, processing, and analysis are provided in the Supporting Information, including Tables S1–S9.

In brief, patients undergoing liver biopsy for diagnostic clinical purposes were invited to also undergo FNA of the liver at the same time, as well as peripheral blood sampling. Mononuclear cells were isolated from the FNAs and peripheral blood and compared using flow cytometry. Furthermore, patients with PSC underwent FNA alone (without liver biopsy) as well as peripheral blood sampling, and again mononuclear cells were compared using flow cytometry. Safety outcomes were compared between patients undergoing FNA alone and FNA plus concurrent liver biopsy. Mononuclear cells from liver resections being done for clinical purposes were also compared with matched peripheral blood. Finally, a sub‐analysis was performed of scRNAseq on matched FNAs and PBMCs from patients with either PSC or MASLD.

## 3 Results

### 3.1 Baseline Demographics

**FIGURE 1:**
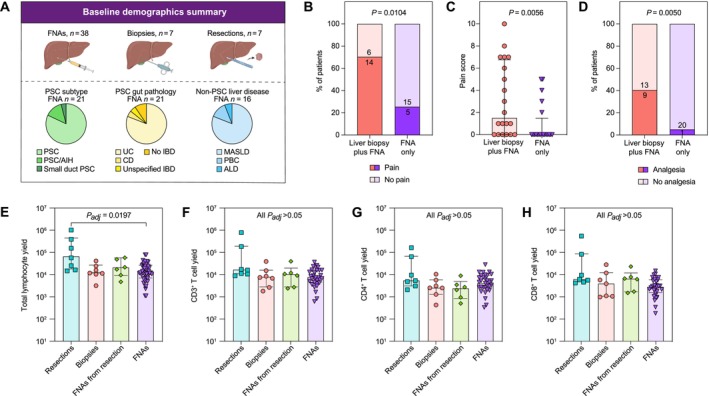
Patient baseline demographics, post‐procedure pain and hepatic lymphoid cell yields by liver sampling technique. (A) Schematic of patient numbers by liver sampling technique. Pie charts summarise the disease type of patients from whom FNA samples were obtained. (B) Percent and numbers of patients experiencing pain after liver biopsy with concomitant FNA or FNA only.*p‐value estimated using Fisher's test (two‐sided). (C) Pain score out of 10 experienced patients 1 h after liver sampling procedure (either liver biopsy with concomitant FNA or FNA only).*Medians with SEM shown. *p*‐value estimated using a two‐tailed Mann–Whitney test. (D) Percent and number of patients requiring analgesia after liver biopsy with concomitant FNA or FNA only. *p*‐value estimated using Fisher's test (two‐sided). Total hepatic lymphocyte (E), CD3^+^ T (F), CD4^+^ T (G) and CD8^+^ T (H) cell yields by liver sampling method, as measured by flow cytometry (Kruskall‐Wallis test performed for multiple comparisons). Sampling methods included resections (*n* = 7), biopsies (*n* = 7), FNAs from resections (*n* = 6), and FNAs (*n* = 31). For (E–H), Medians with SEM shown; *p*‐value estimated using Kruskall–Wallis test with Dunn's multiple comparisons. AIH, autoimmune hepatitis; ALD, alcohol‐related liver disease; CD, Crohn's disease; IBD, inflammatory bowel disease; MASLD, metabolic‐associated steatotic liver disease; *P*_*adj*_, *p*‐value adjusted for multiple comparisons; PBC, primary biliary cholangitis; PSC, primary sclerosing cholangitis; UC, ulcerative colitis. *Pain scores were available for 40/43 patients undergoing a liver sampling procedure. Note that 5 patients underwent FNA on two separate occasions, with pain score included on both occasions (hence representing 38 individual patients).

Liver‐derived samples were collected from 52 patients: 43 FNAs from 38 patients (22 of whom also had a concomitant liver biopsy, 16 patients had FNA alone with no concomitant liver biopsy); 7 patients from whom liver biopsies were taken with no concomitant FNAs; and 7 patients who had liver resections (Figure 1A; Table 1). Paired peripheral blood mononuclear cells (PBMCs) from the same individual at the same time point were available for all FNA samples. 5/38 patients (13%) undergoing FNA had an inadequate initial sample, so underwent a second FNA at a later time point.

**TABLE 1:** Baseline demographics of patients undergoing liver FNA, biopsy, and resection.

Variable	FNA^a^ (*n* = 38)	Liver biopsy (*n* = 7)	Liver resection (= 7)	*p*
Age, years, median (IQR)	47.9 (34.0–61.0)	31.5 (25.2–39.2)	65.3 (51.8–70.9)	0.002
Male sex, *n* (%)	23 (61%)	3 (43%)	5 (71%)	0.542
Type of liver disease^b^				
PSC	21 (55%)	7 (100%)	0 (0%)	0.002
MASLD	13 (34%)	1 (14%)^c^	5 (71%)	
PBC	2 (5%)	0 (0%)	0 (0%)	
ALD	1 (3%)	0 (0%)	0 (0%)	
AIH	0 (0%)	1 (14%)^c^	0 (0%)	
Non‐specific changes on histology	1 (3%)	0 (0%)	2 (29%)	
Type of PSC, *n* (% of n with PSC)				
Classical PSC	17 (81%)	6 (86%)	n/a	0.772
PSC/AIH overlap	3 (14%)	1 (14%)^c^		
Small Duct‐PSC	1 (5%)	1 (14%)^c^		
Type of IBD, *n* (% of *n* with PSC)				
Ulcerative colitis	17 (80%)	5 (71%)	n/a	0.604
Crohn's disease	1 (5%)	0 (0%)		
Unspecified IBD	1 (5%)	0 (0%)		
No IBD	2 (10%)	2 (29%)		
UDCA at baseline, *n* (% of *n* with PSC)	12 (57%)	n.d.	n/a	—
Concomitant liver biopsy, *n* (%)	22 (58%)	n/a	n/a	—
Fibroscan LSM, kPa, median (IQR)^d^	11.5 (5.9–16.2)	n.d.	n.d.	—

Abbreviations: AIH, autoimmune hepatitis; ALD, alcohol‐related liver disease; IBD, inflammatory bowel disease; LSM, liver stiffness measurement; MASLD, metabolic dysfunction‐associated steatotic liver disease; n.d., no data; n/a, not applicable; PBC, primary biliary cholangitis; PSC, primary sclerosing cholangitis; UDCA, ursodeoxycholic acid.

^a^ Twenty‐two patients undergoing FNA had concomitant liver biopsy, while 16 patients underwent FNA alone.

^b^ For patients who had a liver resection—the resection was from background non‐cancerous tissue.

^c^ This represents the same single patient with small duct‐PSC, AIH, and steatosis all on the one liver biopsy.

^d^ Fibroscan LSM was available for 28/38 patients.

The patients from whom FNAs were obtained (*n* = 38) had a median age of 47.9 years (interquartile range, IQR = 13.1 years), and 61% were male. Of the 38 patients sampled by FNA, 21 (55%) had PSC, with 81% displaying a classical disease presentation. Eighty‐one percent of PSC patients presented with ulcerative colitis (UC) comorbidity, whilst only 9.5% had no inflammatory bowel disease. Of the non‐PSC patients who were sampled by FNA, the majority (13/38, 34.2%) were patients with MASLD.

All seven patients undergoing liver biopsy for research purposes (i.e., not concomintantly with FNA) had PSC, with one of these patients having SD‐PSC, MASLD, and autoimmune hepatitis concomitantly. Meanwhile, the majority of patients who had liver resections had underlying MASLD.

Across the different liver sampling methods (FNA, biopsy and resection), there were no significant differences in the mean age, sex distribution, or baseline blood measurements of patients (Table 1).

### 3.2 High Safety and Patient Acceptability of FNA

Pain score out of 10 was obtained at 1 h post‐procedure in patients undergoing FNA with concomitant liver biopsy and patients undergoing FNA alone (available for 40/43 patients). Of those who underwent both FNA and concomitant liver biopsy (*n* = 22), 70% experienced pain post‐procedure pain score ≥ 1, compared with only 25% of the FNA‐only patients (*p* = 0.0104; Figure 1B). The median pain score was higher in the liver biopsy/FNA cohort (1.5, IQR = 0.0–6.8) than in the FNA‐only cohort (0.0, IQR = 0.0–1.5, *p* = 0.0056; Figure 1C).

One patient (4.8%) required analgesia in the FNA‐only cohort versus 9 patients (40.9%) in the liver biopsy/FNA cohort (*p* = 0.0050; Figure 1D). Of those that required analgesia, most required paracetamol only (7/10), and 3 patients required subsequent opiate analgesia (1 in the FNA‐only cohort, 2 in the liver biopsy/FNA cohort). One serious adverse event occurred within the liver biopsy/FNA cohort—an intrahepatic haematoma causing significant pain and requiring overnight hospital admission. This was managed conservatively with observation and analgesia, and the patient was discharged the following day with no long‐term sequelae. There were no serious adverse events in the FNA‐only cohort.

Qualitatively, FNA was well received by patients. The majority of patients were willing to undergo FNA after reading the patient information sheet. Patients with PSC were invited to undergo a second FNA. Of the 17/21 who could be contacted, 15 (88%) were willing to undergo a second FNA in the future.

### 3.3 FNA Lymphoid Cell Yields Are Comparable to Those From Biopsies

Given the safety and patient acceptability of FNAs, we next sought to assess via flow cytometry how the cell yields from FNAs compared to that of other liver sampling techniques, including biopsies and resections (Figure 1E–H), noting that approximately 50 mg of biopsy material and 50 g of resection material were processed per sample. It should be noted that only 31/38 paired FNA/PBMC samples were used, as there were insufficient cells for flow cytometry analysis in 6/38 FNA samples (16%) and one set of samples was inadvertently discarded through a laboratory accident. All seven liver resection samples and all seven liver biopsy samples were analysed via flow cytometry. It should also be noted that an FNA was taken from the ex vivo resection sample in 6 cases and treated the same as FNAs taken percutaneously, as a comparison for yield.

Using flow cytometry (Figure S1), the median yield of the total live, single cells from FNAs was estimated to be 34 664 cells (IQR = 11 895–127 658 cells), which was not significantly different to the yield from biopsies (median = 42 062) or resections (median = 527 050). Similarly, no differences (all *P*_*adj*_ > 0.05) were observed in the yield of non‐immune (FNA median = 2436 cells, IQR = 1342–49 608 cells) or immune cells (FNA median = 23 213 cells, IQR = 9497–39 627 cells) or in the percentage of immune cells out of the total live, single cells (FNA median = 86.17%, IQR = 50.52%–95.36%; Table S2).

The median lymphocyte yield from FNAs was estimated to be 12 333 cells (IQR = 6392–24 205 cells), which was not significantly different to the yield from biopsies or from FNAs derived from resections, although the resections themselves had a significantly higher yield with a median of 57 057 cells (IQR = 16 227–392 624 cells; *P*_*adj*_ = 0.0197; Figure 1E). Despite this difference for total lymphocytes, the yield of total T cells and of CD4^+^ and CD8^+^ T cells was not significantly different across the sampling methods tested, with FNAs yielding a median number of 7547 T cells (IQR = 4456–15 910 cells), 4352 CD4^+^ T cells (IQR = 2450–8818 cells), and 2827 CD8^+^ T cells (IQR = 1558–5825 cells), respectively (Figure 1F–H).

### 3.4 Flow Cytometric Comparison of Paired Liver FNAs and PBMCs Reveals Enrichment of Activated, Memory, and Innate Lymphoid Populations

**FIGURE 2:**
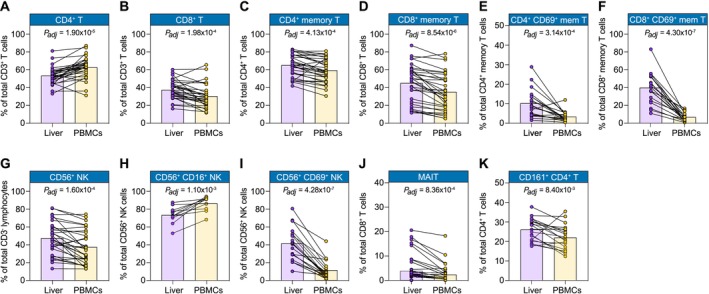
Comparison of liver FNA and blood‐derived lymphocyte subset frequencies by flow cytometric analysis. Percent of CD4^+^ T (A), CD8^+^ T (B), CD4^+^ memory T (C), CD8^+^ memory T (D), CD4^+^ CD69^+^ memory T (E), CD8^+^ CD69^+^ memory T (F), CD56^+^ NK (G), CD56^+^ CD16^+^ NK (H), CD56^+^ CD69^+^ NK (I), MAIT (J), and CD161^+^ CD4^+^ T (K) cells in paired liver FNA versus peripheral blood samples. (A–D) *N* = 31; (E, F) *N* = 19; (G) *N* = 30; (H) *N* = 11; (I–K) *N* = 19. Approximately half of patients had PSC, the remainder having other liver aetiologies (see Table 1). Purple symbols correspond to the liver FNA samples; yellow symbols correspond to the matched PBMCs. Pairs of samples are connected by lines. Bars show the median cell frequency (as a percentage) for each tissue compartment. Adjusted *p*‐values estimated using a two‐tailed Wilcoxon matched‐pairs signed rank test with a Benjamini‐Hochberg correction for multiple testing. MAIT, mucosal‐associated invariant T; mem, memory; NK, natural killer; PBMCs, peripheral blood mononuclear cells.

The cell composition of CD45^+^ mononuclear cells was compared between samples derived from liver FNA with paired PBMCs from the same individual using flow cytometry (Figure 2; Figure S1). When analysing the T‐cell populations, there was a lower median proportion of CD4^+^ T cells (out of the total CD3^+^ T cells) in liver FNA samples compared with paired PBMCs (53.1%, IQR = 47.4%–58.0% vs. 63.4%, IQR = 56.7%–67.9%, *P*_*adj*_ = 1.90 × 10^−5^; Figure 2A). Consistent with a tissue‐specific phenotype, in FNA samples as compared with paired PBMCs, there was an enrichment of CD8^+^ T cells, memory CD4^+^ and CD8^+^ T cells (CD45RA^−^), and in particular CD69^+^ memory CD4^+^ and CD8^+^ T cells (all *P*_*adj*_ < 0.05; Figure 2B–F).

There was also a higher median proportion of NK cells (CD56^+^) amongst the CD3^−^ lymphoid populations in FNA samples (46.1%, IQR = 33.5%–60.2%) compared with paired PBMCs (34.5%, IQR = 21.8%–54.4%; *P*_*adj*_ = 1.60 × 10^−4^; Figure 2G). Of these NK cells, in the FNA samples a lower proportion was CD16^+^ (72.8%, IQR = 70.8%–78.8%) compared to paired PBMCs (91.0%, IQR = 80.4%–92.5%; *P*_*adj*_ = 1.10 × 10^−3^; Figure 2H). Loss of CD16^+^ expression on NK cells is associated with cell activation and interferon (IFN)‐γ production [15], and may indicate a distinct NK cell population within FNA samples. In keeping with the increased activation of NK cells as indicated by the loss of CD16 in the liver compared to the peripheral blood, a higher median proportion of NK cells was CD69^+^ (41.9%, IQR = 29.0%–52.2%) compared to the PBMCs (6.52%, IQR = 4.8%–16.1%; *P*_*adj*_ = 4.28 × 10^−7^; Figure 2I).

Mucosal‐associated invariant T cells (MAITs) have been shown to be enriched in the liver as compared with the peripheral blood, both in health and in chronic inflammatory liver diseases [16, 17, 18]. A higher median proportion of CD8^+^ T cells were MAITs (CD161^++^ Vα7.2^+^) in FNA samples (3.7%, IQR = 2.1%–14.3%) compared with paired PBMCs (2.3%, IQR = 1.0%–4.9%; *P*_*adj*_ = 8.36 × 10^−4^; Figure 2J). There are limited data on the proportion and function of CD161^+^ CD4^+^ T cells within the liver; however, we also found an enrichment of these cells in FNA samples (median = 26.2%, IQR = 20.8%–30.4%) as compared with paired PBMCs (21.0%, IQR = 15.9%–26.3%, *P*_*adj*_ = 8.40 × 10^−3^; Figure 2K).

Whilst not within the primary scope of current analyses, FNAs were also interrogated as to whether there was a difference in CD4^+^ and CD8^+^ T cell surface expression of particular markers with respect to PSC versus non‐PSC conditions (Figures S2 and S3). There was no difference in the proportion of these T‐cell subsets with relation to CC‐chemokine receptor 9 (CCR9), β7 (as a surrogate for α4β7), or CD161 (Figures S4 and S5). However, a higher proportion of activation marker CD69 amongst CD8^+^ memory T cells was observed in PSC compared to non‐PSC (Figure S5).

### 3.5 High‐Resolution Cell Cluster Identification by scRNA‐Seq Delineates Cell States Across Immune Compartments in the Liver and Blood

**FIGURE 3:**
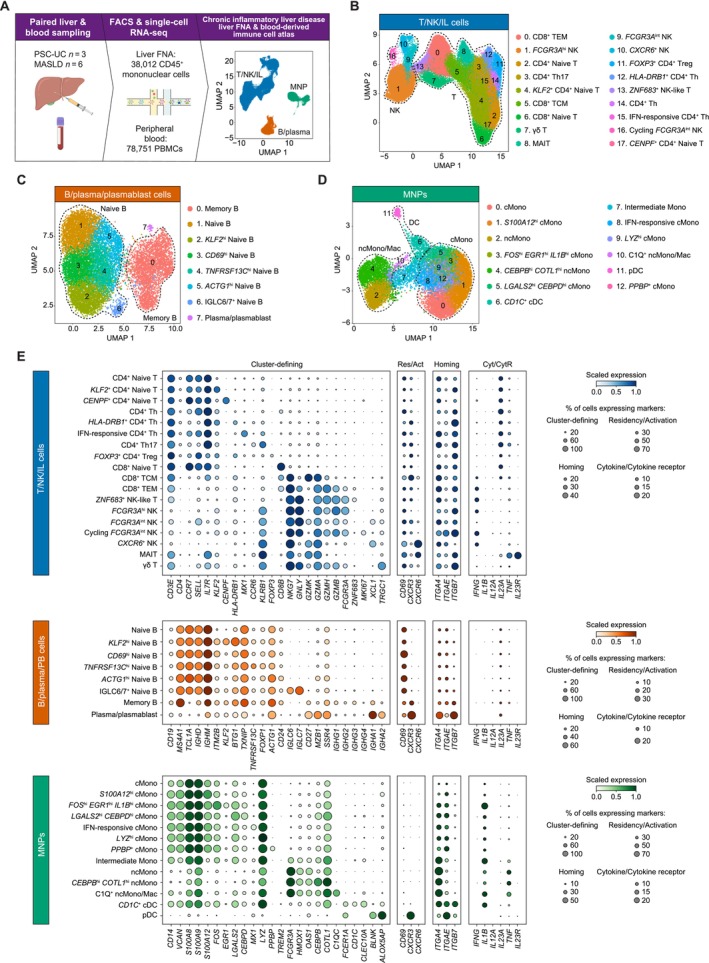
Paired liver FNA and blood‐derived immune cell atlas of non‐infectious, chronic inflammatory liver disease. (A) Schematic of single‐cell RNA‐seq study design, indicating the number of patient samples analysed and the number of liver FNA and peripheral blood cells recovered after scRNA‐seq. T/NK/IL (B), B/plasma/plasmablast (C), and MNP (D) cell clusters identified. For each of these three immune cell compartments, UMAP (uniform manifold approximation and projection) plots are shown depicting the cell cluster distributions and annotating the main cell types that the clusters belong to (denoted by the dotted lines). Individual cell states are numbered, with their respective annotations shown to the left of the plots. (E) Scaled gene expression of key cell markers in the identified cell clusters for each of the three main immune cell compartments. Gene expression is shown per cell state as a dot plot where dot size indicates the proportion of cells expressing the marker gene and dot colour indicates the level of mean expression. For each cell state, cluster‐defining markers, markers of tissue residency (Res) or activation (Act), tissue homing markers, and key cytokines (Cyt) and cytokine receptors (CytR) are plotted. Act, activation; cDC, conventional dendritic cells; cMono, classical monocytes; Cyt, cytokine; CytR, cytokine receptor; DC, dendritic cell; FNA, fine needle aspirate; hi, high; IFN, interferon; IL, innate lymphoid; int., intermediate; MAIT, mucosal‐associated invariant T; MASLD, metabolic dysfunction‐associated steatotic liver disease; MNP, mononuclear phagocyte; Mono, monocyte; ncMono/Mac, non‐classical monocyte/macrophage; NK, natural killer; PBMC, peripheral blood mononuclear cell; pDC, plasmacytoid dendritic cell; PSC‐UC, primary sclerosing cholangitis‐ulcerative colitis; Res, residency; RNA‐seq, RNA sequencing; TCM, central memory T; TEM, effector memory T; Th, CD4^+^ helper T; Treg, CD4^+^ regulatory T; UMAP, uniform manifold approximation and projection.

To further characterise the immune cell states found in liver FNAs compared to matched PBMCs, paired samples were obtained from 9 patients, 3 with PSC and comorbid UC (PSC‐UC) and 6 with MASLD (Table S4). All samples were sorted to obtain live CD45^+^ mononuclear cells, and 10× Genomics 3′ scRNA‐seq was performed, yielding 38 012 and 78 751 high‐quality transcriptomes from the FNAs and PBMCs, respectively (Figure 3A; Table S5). Integration and clustering of all samples and cells was performed to facilitate FNA and PBMC comparisons, and across the tissue compartments 18 T/NK (Figure 3B), 8 B/plasma/plasmablast (Figure 3C) and 13 mononuclear phagocyte (MNP; Figure 3D) cell states were identified. Cell states were annotated based on cluster‐defining markers and markers indicative of tissue residency/activation and homing, and cytokine receptor expression (Figure 3E).

Within the T/NK compartment multiple naïve and memory CD4^+^ and CD8^+^ T, NK, MAIT and γδ T cell states were identified. The IFN‐responsive CD4^+^ T helper (Th), Th17, and CD8^+^ T effector memory (TEM) cells had the highest expression of the gut‐homing integrin genes *ITGA4* and *ITGB7*, which may be implicated in trafficking of gut‐derived T cells into the liver. *CXCR6* ^+^ NK and MAIT cells, and to a lesser extent CD8^+^ T central memory (TCM) cells had the highest expression of the *CXCR6* liver residency marker (Figure 3E). Within the B/plasma/plasmablast compartment naïve and memory B cell subsets and plasma/plasmablast cells with high IgA gene expression were discernable (Figure 3F). The MNP compartment included classical *CD14* ^+^ and non‐classical *FCGR3A* ^+^ monocyte/macrophage populations (cMono and ncMono/Mac, respectively), with the latter having the highest expression of tumour necrosis factor (*TNF*) amongst the MNPs, as well as *CD1C* ^+^ conventional and plasmacytoid dendritic cells (*CD1C* ^+^ cDCs and pDCs, respectively; Figure 3G).

### 3.6 Liver NK and T‐Cell State Differential Abundances Relative to Matched PBMCs Demonstrate the Capacity of FNA to Sample Liver‐Enriched Cells

**FIGURE 4:**
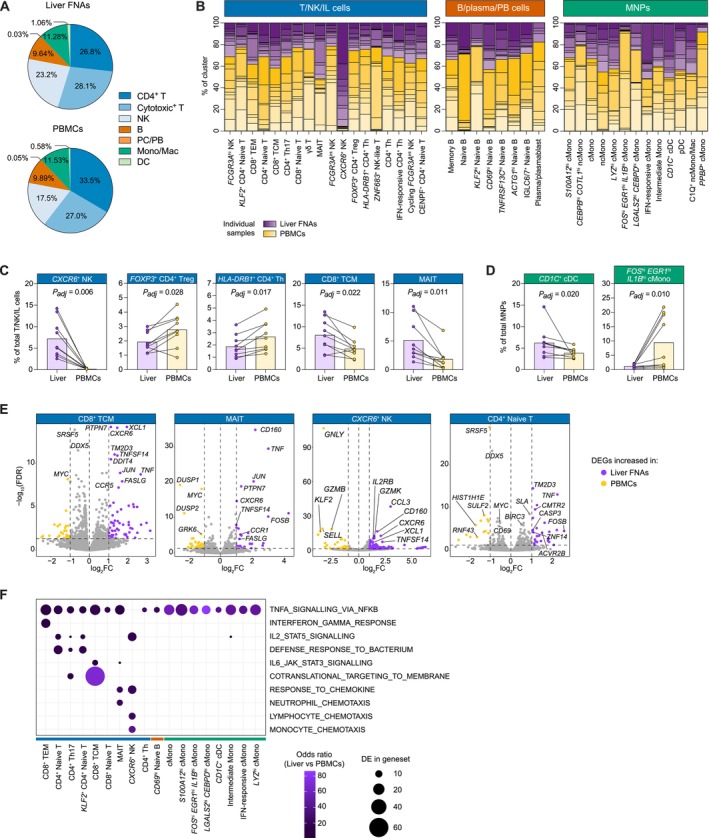
Single‐cell RNA‐seq differential abundance and gene expression analyses comparing paired liver FNA and blood‐derived immune cells. (A) Pie charts showing the average percent of the main immune cell types found in liver FNAs (top) and PBMCs (bottom). (B) Stacked bar plots showing the percent of each cluster represented by individual samples (liver FNAs shown in purple; PBMCs shown in yellow). Bars correspond to cell clusters, whilst stacks correspond to the individual samples. T/NK/IL cell (C) and MNP cell (D) clusters with a significant differential abundance when comparing paired liver FNA and PBMC samples. Purple symbols correspond to the liver FNA samples; yellow symbols correspond to the matched PBMCs. Pairs of samples are connected by lines. Bars show the median cell frequency (as a percentage) for each tissue compartment. Adjusted *P*‐values estimated using a two‐tailed Wilcoxon matched‐pairs signed rank test with a Benjamini‐Hochberg correction for multiple testing. (E) Differential gene expression analysis of selected T/NK‐cell subsets when comparing paired liver FNA and PBMC samples. Left and right vertical dotted lines correspond to the log_2_FC thresholds used and the horizontal dotted line denotes the adjusted signifance threshold (FDR < 0.05). Purple and yellow symbols denote genes with statistically significantly increased expression in the liver and blood, respectively. (F) Pathways differentially enriched between paired liver FNA and PBMC samples across immune cell clusters (determined using a Fisher test and gene set enrichment analysis). Dot plot shows cell clusters on the *x*‐axis and key pathways on the *y*‐axis. Dot size corresponds to the number of differentially expressed genes in the geneset; dot colour shows the odds ratio of pathway enrichment, where an odds ratio of > 1 indicates enrichment in the liver versus PBMCs. cDC, conventional dendritic cells; cMono, classical monocytes; DC, dendritic cell; DEG, differentially expressed gene; FC, fold change; FDR, false discovery rate; FNA, fine needle aspirate; hi, high; IFN, interferon; IL, innate lymphoid; int., intermediate; MAIT, mucosal‐associated invariant T; MNP, mononuclear phagocyte; Mono, monocyte; Mono/Mac, monocyte/macrophage; ncMono/Mac, non‐classical monocyte/macrophage; NK, natural killer; *P*_*adj*_, P‐value adjusted for multiple comparisons; PBMC, peripheral blood mononuclear cell; PB, plasmablasts; PC, plasma cells; pDC, plasmacytoid dendritic cell; TCM, central memory T; TEM, effector memory T; Th, CD4^+^ helper T; Treg, CD4^+^ regulatory T.

Comparing the proportions of the main immune cell types in the FNA samples to those of the matched PBMCs, the liver had a relatively higher average proportion of NK cells (23.2% vs. 17.5%) and a lower proportion of CD4^+^ T cells (26.8% vs. 33.5%), whilst the other cell types were more comparable (Figure 4A). Differential abundance analysis of individual cell states, which were well represented across all patients (Figure 4B; Table S5), demonstrated that the higher proportion of NK cells in the liver FNAs compared to the PBMCs was due to the presence of the *CXCR6* ^+^ NK cells (*P*_*adj*_ = 0.006; Figure 4C). These cells have been previously described as a liver‐specific cell type that is adapted for hepatic tolerance and inducible anti‐viral immunity [19, 20]. The detection of this cell state demonstrates the capacity of the FNA technique to sample liver‐resident cell types. The relative decrease in the proportion of liver versus PBMC CD4^+^ T cells was predominantly driven by the lower frequency of *FOXP3* ^+^ CD4^+^ regulatory T cells (Tregs) and *HLA‐DRB1* ^+^ CD4^+^ Th cells in the liver (*P*_*adj*_ = 0.028 and *P*_*adj*_ = 0.017, respectively; Figure 4C). Further to the differences in NK and CD4^+^ T cell state abundances between the liver and PBMCs, differences were also observed in the cytotoxic T cell compartment. Specifically, CD8^+^ TCM and MAIT cells were enriched in the liver FNAs compared to PBMCs (*P*_*adj*_ = 0.022 and *P*_*adj*_ = 0.011, respectively; Figure 4C), consistent with their expression of liver residency markers. No abundance differences were observed for the B/plasma/plasmablast cell states, but differences were discernible within the myeloid compartment. *CD1C* ^+^ cDC and *FOS* ^*hi*^ *EGR1* ^*hi*^ *IL1B* ^*hi*^ cMono cell frequencies were higher and lower, respectively, in the liver FNAs versus the paired PBMCs (*P*_*adj*_ = 0.020 and *P*_*adj*_ = 0.010, respectively; Figure 4D). Cell state frequencies were not significantly different within the liver or amongst PBMCs when contrasting the PSC‐UC with the MASLD patients (cell counts per cell state, patient and tissue are provided in Table S5).

### 3.7 Differential Gene Expression and Gene Set Enrichment Analyses Demonstrate Cytokine and Chemokine Signalling as Enhanced in Liver FNAs Relative to Matched PBMCs

Investigating gene expression differences across cell types in the FNAs as compared with the paired PBMCs revealed increased expression of genes implicated in cytokine and chemokine signalling and cell activation, mainly in the T‐cell compartment in the liver (Figure 4E; Tables S6 and S7). The most prominent differences in gene expression were observed for the CD8^+^ TCM cells, where 124 genes showed significantly higher expression in the liver FNAs as opposed to 88 genes that were higher in PBMCs. The differentially expressed genes (DEGs) that were higher in the liver for this cell state included TNF superfamily members *TNF*, *TNFSF14*, *FASLG* and *CD160*; interferon genes *IFNG* and *IFNL1*; chemokines and their receptors such as *XCL1*, *CCL3*, *CXCR6*, and *CCR1*; and the immune activation transcription factors *JUN* and *FOSB*. For the MAIT cells, 28 genes were increased in the liver FNAs, and similarly to the CD8^+^ TCM cells, these included *TNF*, *FASLG*, *CD160*, *CXCR6*, *CCR1*, *JUN* and *FOSB*. Expression of the *PDCD1* gene, which encodes the checkpoint molecule PD‐1 that is indicative of immune cell activation or exhaustion, was also increased in the liver MAITs. Chemokines and their receptors including *XCL1*, *CCL3*, and *CXCR6*, and genes such as *CD160* and *FOSB* were also more highly expressed in *CXCR6* ^+^ NK cells in the liver, where 200 genes were significantly increased, as opposed to 34 genes in the blood. DEGs were also found for the CD4^+^ naïve T cells between the patient liver and blood, including *TNF* and *FOSB*, which were increased in the hepatic cells, suggesting that these cells have begun to acquire a more activated state (Figure 4E; Table S6).

To further assess cell state differences in gene expression between the liver FNAs and PBMCs, gene set and pathway enrichment analysis was performed (Figure 4F; Table S7). NFκB‐mediated TNF signalling was increased in liver FNAs compared to PBMCs for 17 different T, B and MNP cell states, including naïve T and B cells, CD8^+^ TEM and TCM cells, CD4^+^ Th17 cells, multiple cMono cell states and *CD1C* ^+^ cDCs. Amongst the CD4^+^ T cell states, the hepatic naïve and Th17 cells showed an enrichment for the IL‐2 signalling pathway and for a defence response against bacteria, whilst amongst the CD8^+^ T cells, the liver TEM cells showed increased expression of genes involved in the response to IFNγ and the TCM cells were particularly enriched for genes implicated in translation. The MAIT cells and the *CXCR6* ^+^ NK cells in the FNAs were enriched for immune cell chemotaxis pathways, indicating that this may represent a key function of innate‐like lymphoid cells with the patient liver (Figure 4F; Table S7). Notably, the DEGs and the enriched gene sets and pathways were well‐represented across the PSC‐UC and MASLD patients. Strong expression differences were not observed between the patient groups within the FNAs or the PBMCs (Table S6). Across all cell states, 94 genes were differentially expressed in the liver in PSC‐UC versus MASLD, with 32 of these being increased in PSC‐UC, and including HLA class II genes and genes implicated in inflammatory responses (e.g., *S100B*), whilst genes increased in MASLD included chemokines such as *CCL3L1*. Similar genes were differentially expressed in the PBMCs, where 69 and 192 genes were increased in PSC‐UC and MASLD, respectively (Table S6).

### 3.8 Cell–Cell Interactions Between Immune Cell States in the Liver Are Driven by Myeloid Cells and Are Enriched for Cytokine and Chemokine Ligand‐Receptor Pairs

**FIGURE 5:**
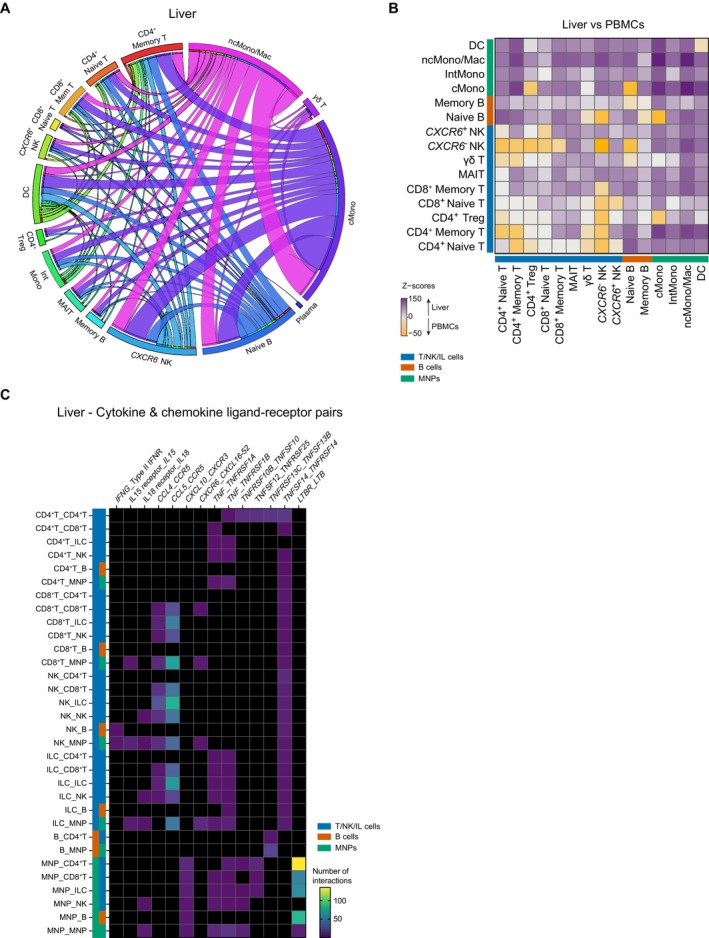
Cell–cell interactions inferred from scRNA‐seq of paired liver FNA and blood‐derived immune cells. (A) Circos plot of cell–cell interactions inferred between the main immune cell types found in liver FNA samples. Cell–cell ligand‐receptor interactions were inferred using CellPhoneDB (www.cellphonedb.org). The lower cutoff for expression proportion of any ligand or receptor in a cell type was set to 10%, and the number of permutations was set to 1000. Annotated cell types correspond to ‘senders’ and are each also denoted by a different colour. Ribbons are coloured by the sender cell type and ribbon width depicts the relative number of inferred cell–cell interactions for any sender and receiver cell type pair. (B) Heatmap showing Z‐scores of the number of cell–cell interactions enriched in liver FNA as compared with blood‐derived immune cells. Purple and yellow scores cell–cell interactions higher in liver and blood, respectively. (C) Specific cytokine and chemokine ligand‐receptor pairs (*x*‐axis) enriched in liver FNA immune cells (*y*‐axis, where the first cell type noted expressed the ligand and the second cell type expressed the receptor). cMono, classical monocytes; DC, dendritic cell; ILC, innate lymphoid cell; IntMono, intermediate monocyte; MAIT, mucosal‐associated invariant T; Mem, memory; MNP, mononuclear phagocyte; ncMono/Mac, non‐classical monocyte/macrophage; NK, natural killer; PBMC, peripheral blood mononuclear cell; Treg, CD4^+^ regulatory T.

Given the differences in gene expression observed between the matched FNAs and PBMCs, we reasoned that these differences may extend to immune cell–cell interactions when comparing the two tissue compartments (Table S8). Cell–cell interactions were inferred using CellphoneDB. In the liver, these interactions predominantly mapped to the myeloid compartment and included intra‐myeloid interactions and interactions between the myeloid cells and lymphocytes (Figure 5A). Constructing a Z‐score to help quantify the relative abundance of cell–cell interactions in the liver FNAs relative to the blood similarly demonstrated the enrichment of myeloid cell interactions in the liver, as well as a number of intra‐lymphocyte interactions, particularly involving CD8^+^ memory T, MAIT, and B cells (Figure 5B).

Investigating the nature and distribution of cytokine, chemokine and TNF superfamily ligand‐receptor pairs underpinning the liver immune cell–cell interactome showed a prominent role for *CCL5‐CCR5* and *CCL4‐CCR5* in interactions between CD8^+^ T cells, NK cells and innate lymphoid cells and multiple other immune cell types (Figure 5C). Interactions between *TNF* and its receptors were observed between CD4^+^ T cells, innate lymphoid cells and myeloid cells, largely in keeping with the observed increased NFκB‐mediated TNF signalling in liver FNAs (Figure 4F). *LTBR‐LTB* interactions were particularly increased in myeloid cells. Intriguingly, in the liver, signalling triggered by lymphotoxin B (encoded by *LTB*) has been implicated in repair, regeneration and carcinogenesis, noting that both PSC and MASLD patients have an increased risk for liver cancer [13, 21, 22, 23].

## 4 Discussion

The increasing global incidence and prevalence of acute and chronic liver disease pose a substantial healthcare burden in the absence of improved advanced therapeutic options, beyond liver transplantation, in late‐stage disease. There is a fundamental need to develop approaches that provide early insights into pathophysiology and allow for longitudinal sampling over time. Blood sampling is minimally invasive but poorly represents tissue‐resident immunity. FNA sampling of the liver is a minimally invasive technique for acquisition of cells present at the site of pathology [4]. This enables direct interrogation of the local cellular and molecular processes implicated in disease development and progression, and furthermore allows investigation of the tissue‐specific effects of treatment.

This technique has been successfully employed in the context of chronic viral hepatitis B and C infections to demonstrate distinct liver‐resident populations, multiple layers of innate immune regulation, and persistent immune modifications after therapy [10, 11, 12, 24, 25, 26]. This is the first study employing FNA sampling of the liver as a means of successfully interrogating the hepatic immune landscape via scRNA‐seq in non‐viral pathologies—in this case, PSC and MASLD.

In this study, we describe that the technique of FNA is safe and well‐tolerated. Patients experienced minimal pain, with only one in 21 patients requiring any analgesia when FNAs were taken instead of liver biopsies. FNAs are collected using a small diameter needle (22–25 gauge) with a much narrower diameter (0.51 mm) compared to liver core biopsies (16–18 gauge; 1.65–1.27 mm), which likely accounts for the better safety and tolerability profile [10]. Indeed, 88% of patients contacted were happy to undergo a follow‐up FNA procedure.

There is concern that liver FNAs may yield fewer cells (< 100 000) compared to conventional liver biopsies. However, in our study, the total CD3^+^ T cell yield from FNAs was comparable to those obtained with resections or biopsies, suggesting that FNA has the ability to enable T‐cell profiling to an analogous degree. This was the case when there were sufficient cells for flow cytometric analysis, noting that there were insufficient cells in 16% of cases from FNA. It should be noted, however, that all seven patients undergoing liver biopsy for experimental analysis had PSC, with no liver biopsy research specimens from patients with MASLD alone. As the lymphocyte infiltration profile may be different between PSC and MASLD, comparisons between liver biopsy and FNA should be restricted to the PSC population, and cannot be generalised to MASLD. It is important to note, however, that FNA was safely conducted in patients with MASLD, suggesting an important tool with the potential to reveal further insights into the immune environment of the liver in patients with MASLD in the future.

Assessment of the relative abundance of T and NK cell subsets by both flow cytometry and scRNA‐seq revealed concordance between the two methods with respect to the lymphoid cell populations enriched in the liver FNAs compared to PBMCs. ScRNA‐seq analyses enabled a more extensive molecular characterisation of these cell populations, demonstrating the capacity of FNA to sample liver‐resident immune cell types including *CXCR6* ^+^ NK cells and CD8^+^ memory T cells and MAIT cells enriched for tissue residency markers (*CXCR6*, *CD69*).

Further to the cell abundance differences captured in liver FNA immune cells compared with PBMCs, differentially expressed genes could also be identified in the T‐cell compartment. These included *TNF* and other TNF superfamily member genes (e.g., *TNF*, *TNFSF14*, *FASLG*) and *CD160*, interferon genes, chemokines and their receptors (e.g., *XCL1*, *CCL3*, *CXCR6*, and *CCR1*), and transcription factors implicated in immune activation (e.g., *JUN, FOSB*).

The differences were further supported by gene set enrichment analysis showing an enrichment of IFN‐response and of IL‐2 and IL‐6 signalling genes in T cells; of chemotaxis signatures in MAITs and *CXCR6* ^+^ NK cells; and of TNF signalling across multiple lymphoid and myeloid cell states. Myeloid cell interactions constituted the greatest proportion of inferred cell–cell interactions in the liver and were higher compared with the interactions within the PBMCs. In keeping with the differential gene expression and the pathway enrichment analyses, several cytokine, chemokine, and TNF superfamily ligand receptor pairs were found to be increased in the liver, including *CCL4, CCL5, TNF*, *TNFSF14*, and *LTB*.

Historically, sorted populations of immune cells have been used to help understand disease pathogenesis in PSC, reporting a variety of immune cells involved in disease development and progression, including CD4^+^ T cells, B cells, dendritic cells, macrophages, and neutrophils. FNA can provide key data on immune cell populations, but it does not retain the tissue architecture. Bulk, single‐cell and single‐nucleus sequencing studies and spatial transcriptomics have recently been reported in patients with advanced‐stage PSC demonstrating some early understanding of cellular identity, function and interactions in this complex disease [27, 28]. Furthermore, single‐cell analysis of the colonic tissue in patients with PSC‐IBD has yielded a unique inflammatory transcriptomic signature linked to dysplasia in this population [29].

Partly due to the comorbidity of PSC and IBD, it has been hypothesised that trafficking of lymphocytes from the gut to the liver may contribute to disease pathology. It has been reported that ligands such as a vascular cell adhesion molecule‐1 (VCAM‐1) and mucosal addressin cellular adhesion molecule‐1 (MAdCAM‐1) are upregulated in hepatic inflammation, thereby increasing recruitment of gut‐derived CCR9^+^ α4β7^+^ T cells, which are increased in abundance in liver explants from PSC compared to primary biliary cholangitis (PBC) patients [30, 31]. Assessing our flow cytometry and scRNA‐seq data for the expression of gut‐homing markers (CCR9 and β7 as a surrogate for α4β7) and activation markers (CD161 and CD69) on T cells did not reveal any significant differences between our PSC and non‐PSC patients, with the exception of a higher proportion of CD69^+^ CD8^+^ memory T cells observed by flow cytometry in PSC FNAs. These data may suggest the relevance of CCR9^+^ α4β7^+^ T cells in both our PSC and non‐PSC (predominatly MASLD) patients, in keeping with the previously described relevance of these cells in steatotic liver disease [32].

Collectively, our findings indicate FNA can be used to electively sample and profile the immune landscape of the liver in a variety of liver diseases, including PSC and MASLD, and generate high‐resolution data. By combining FNA and scRNA‐seq, we can provide a single‐cell window into tissue‐specific immune responses with minimal risk. The technique can be utilised to study early‐stage disease, disease progression, and response to treatment [33]. FNAs may be optimised for future large‐scale, high‐resolution analyses of disease pathology both within the liver and other tissues for the granular analysis of health and disease.

## Author Contributions

**Kate Diana Lynch:** design of study, obtained ethics approval, development of experimental techniques, sample acquisition and processing, analysis of results, drafting of manuscript, joint overall responsibility for study. **Fabiola Curion:** analysis of single cell RNA‐seq data, editing of manuscript. **Hing‐Yuen Yeung:** sample acquisition and processing for RNA‐seq work. **Charlotte Rich‐Griffin:** analysis of single cell RNA‐seq data. **Devika Agarwal:** analysis of single cell RNA‐seq data. **Helen Ferry:** design of experimental techniques with relation to flow cytometry and fluorescence‐activated cell sorting (FACS). **Andrew Slater:** sample acquisition. **Emma Louise Culver:** data acquisiton, editing of manuscript. **Roger William Chapman:** design of study, co‐supervision of Kate Diana Lynch during study, editing of manuscript. **Satish Keshav:** design of study, direct supervision of Kate Diana Lynch during study, analysis of results. **Paul Klenerman:** design of study, direct supervision of Kate Diana Lynch during study, analysis of results, editing of manuscript, joint overall responsibility of study. **Calliope Athena Dendrou:** design of study, direct supervision of Hing‐Yuen Yeung during sample acquisition and processing for scRNA‐seq, direct supervision of Fabiola Curion, Charlotte Rich‐Griffin, and Devika Agarwal during data analysis of scRNA‐seq, analysis of results, drafting of manuscript, joint overall responsibility for study.

## Ethics Statement

All samples were obtained from patients with written informed consent under Research Ethics Committee approval (South Central—Oxford B Research Ethics Committee reference 17/SC/0290 and 09/H0603/19).

## Conflicts of Interest

The authors declare no conflicts of interest. Please refer to the accompanying ICMJE disclosure forms for further details.

## Supporting information


**Data S1:** liv70358‐sup‐0001‐Supinfo.docx.

**Table S5:** liv70358‐sup‐0002‐TableS5.xlsx.

**Table S6:** liv70358‐sup‐0003‐TableS6.xlsx.

**Table S7:** liv70358‐sup‐0004‐TableS7.xlsx.

**Table S8:** liv70358‐sup‐0005‐TableS8.xlsx.

## Data Availability

All raw and processed data will be available upon acceptance via Human Cell Atlas, EGA and Zenodo. All code will be available on GitHub upon acceptance of this paper. Supporting Information is available for this paper.
