# Supplementary material for: Navigating the Hepatic Immune Landscape With Fine Needle Aspiration of the Liver—An Emerging Technique
Source: Liver Int. 2025 Oct 21;45(11):e70358. doi: 10.1111/liv.70358 (PMC12539073; doi:10.1111/liv.70358)
Supplement: Supplementary file 1 — Data S1: liv70358‐sup‐0001‐Supinfo.docx. [file LIV-45-0-s005.docx]

***Supplementary Information*:**

**Single-cell profiling of liver inflammation in primary sclerosing cholangitis and steatotic liver disease by fine needle aspiration**

Kate Diana Lynch^1,2,3#^, Fabiola Curion^4-6^, Hing-Yuen Yeung^4^, Charlotte Rich-Griffin^4^, Devika Agarwal^4,7^, Helen Ferry^1^, Andrew Slater^8^, Emma Louise Culver^1^, Roger William Chapman^1^, Satish Keshav^1*^, Paul Klenerman^1^, Calliope Athena Dendrou^4,7#^

**Affiliations:**

^1^Translational Gastroenterology Unit, NDM Experimental Medicine, University of Oxford, Oxford, UK

^2^Faculty of Health and Medical Sciences, University of Adelaide, Adelaide, Australia

^3^Department of Gastroenterology and Hepatology, Royal Adelaide Hospital, Central Adelaide Local Health Network, Adelaide, Australia

^4^Nuffield Department of Medicine, Wellcome Centre for Human Genetics, University of Oxford, Oxford, UK

^5^Institute of Computational Biology, Helmholtz Center Munich, Germany

^6^Department of Mathematics, School of Computation, Information and Technology, Technical University of Munich, Germany

^7^Nuffield Department of Orthopaedics, Rheumatology, and Musculoskeletal Sciences, Kennedy Institute of Rheumatology, University of Oxford, Oxford, UK

^8^Department of Radiology, John Radcliffe Hospital, Oxford University Hospitals NHS Foundation Trust, Oxford, UK

**^#^Corresponding authors:**

Kate Diana Lynch

Department of Gastroenterology and Hepatology

Royal Adelaide Hospital

Port Rd, Adelaide, SA, 5000, AUSTRALIA

Ph: +61 8 7074 2182

Fax: +61 8 7074 6192

Email: [kate.lynch@sa.gov.au](mailto:kate.lynch@sa.gov.au)

Calliope Athena Dendrou

Nuffield Department of Orthopaedics, Rheumatology, and Musculoskeletal Sciences Kennedy Institute of Rheumatology, University of Oxford

Roosevelt Drive, Oxford, OX3 7FY, UK

Ph: +44 791 941 7237

Email: [calliope.dendrou@kennedy.ox.ac.uk](mailto:calliope.dendrou@kennedy.ox.ac.uk)

**Table of Contents:**

**Patients & Methods:**

*Patient recruitment and sample acquisition*

*Patient inclusion and exclusion criteria for FNA acquisition*

*Patient acceptability and safety analysis*

*Isolation of hepatic immune cells from liver biopsies and liver resection tissue*

*Isolation of hepatic immune cells via FNA*

*Peripheral blood mononuclear cell (PBMC) isolation*

*Flow cytometry*

*FACS*

*Sample processing for 10x Genomics single-cell 3’ library generation*

*Single-cell RNA-seq preprocessing and quality control*

*Single-cell RNA-seq integration and clustering*

*Single-cell RNA-seq differential abundance analysis*

*Single-cell RNA-seq differential expression and cell-cell interaction analyses*

*Single-cell RNA-seq data visualisation*

*Statistical analyses*

**Supplementary Tables:**

*Supplementary Table 1*. Antibodies used in this study

*Supplementary Table 2*. Summary statistics of total, non-immune and immune cell yields and the percentage of immune cells by liver sampling method

*Supplementary Table 3*. Software and algorithms used in this study

*Supplementary Table 4*. Baseline demographics of patients whose FNA samples were used for scRNA-seq

*Supplementary Table 9*. Deposited data

**Supplementary Tables provided as separate xls:**

*Supplementary Table 5*. Per sample and per cluster cell counts for scRNA-seq data

*Supplementary Table 6*. Single-cell RNA-seq differential gene expression analyses

*Supplementary Table 7*. Gene set/pathway enrichment analysis of FNA vs PBMC scRNA-seq

*Supplementary Table 8*. Cell-cell interaction analysis of FNA vs PBMC scRNA-seq

**Supplementary Figures:**

*Supplementary Figure 1.* Flow cytometry gating strategy

*Supplementary Figure 2:* CD8^+^ T cell gating for MAITs, CCR9, and β7

*Supplementary Figure 3:* CD4^+^ T cell gating for CD161, CCR9, and β7

*Supplementary Figure 4.* CCR9 and β7 expression on liver-derived T cells in PSC and controls

*Supplementary Figure 5.* CD161 and CD69 expression on liver-derived T cells in PSC and controls

**Patients & Methods:**

*Patient recruitment and sample acquisition*

Non-infectious liver disease patients undergoing ultrasound-guided percutaneous liver biopsy or a liver resection for clinical purposes were invited to take part in the study. Patients with PSC, regardless of whether they were undergoing an intervention or not, were also invited to take part in the study. Patients were identified from existing clinical databases at John Radcliffe Hospital, Oxford, UK, and also, in the setting of PSC, were recruited via advertisement through patient charity, PSC Support, from around the UK.

For patients undergoing liver biopsy, consent was taken to obtain a further single additional pass of the liver biopsy needle to obtain a sample for this research study. For those undergoing liver resection (usually for resection of primary hepatocellular carcinoma without known underlying liver disease, or colorectal cancer metastases) consent was taken to obtain a small section of liver tissue from the edge of the resection specimen (with a weight of approximately 2-17 g) away from the lesion, for research purposes.

Additionally, patients undergoing liver biopsy for clinical purposes were invited to take part in the FNA portion of the study. In this setting, patients gave consent to have a liver FNA performed immediately after their liver biopsy (only the FNA was kept for research). Patients with PSC not undergoing a liver intervention gave consent to undergo a liver FNA alone. Patients with PSC were invited to undergo a second FNA if the initial FNA sample was inadequate. Peripheral blood collection was performed for all patients for PBMC acquisition and analysis on the same day as liver sample collection. Baseline demographics and disease characteristics were collected for all patients recruited.

Patient’s diagnosis, liver biochemistry, and general demographics were collected at baseline. At the time of patient recruitment, the terminology for metabolic dysfunction-associated steatotic liver disease (MASLD) was not in existence, and patients with what was previously known as non-alcoholic fatty liver disease (NAFLD) were among those recruited. For the purposes of this paper and current accepted international terminology, they have been re-classified as MASLD.

*Patient inclusion and exclusion criteria for FNA acquisition*

Inclusion Criteria:

- Participant is willing and able to give informed consent for participation in the study.
- Male or Female, aged 18 years to 75 years.
- Upper abdominal ultrasound within the last 12 months (or, willing to undergo an upper abdominal ultrasound prior to FNA Liver).

PSC Cohort: extra inclusion criteria

- Diagnosed with Primary Sclerosing Cholangitis according to standard clinical criteria for >3 months.

Control cohort: extra inclusion criteria

- Patients with suspected or definite chronic liver disease.
- Scheduled to have a liver biopsy for clinical purposes during the study period.

Exclusion Criteria:

The participant may not enter the study if ANY of the following apply:

- Unable to consent.
- Significant comorbid medical condition(s) which may increase the risk of an FNA Liver.
- Blood tests within previous 3 months which would increase risk of bleeding:
  - INR>1.3 or prothrombin time test>16 seconds.
  - Platelet count <100 x 10^3^/L.
- Known hepatobiliary malignancy.
- Clinical suspicion or evidence of ascites.
- Use of an oral anticoagulant or antiplatelet agent.
- Pregnancy.
- Any concern by the investigator regarding the safe participation of the patient in the study; or investigator’s consideration, for any other reason, that a patient is inappropriate for participation in the study.

*Patient acceptability and safety analysis*

Pain score out of 10 was obtained at 1 h post-procedure in patients undergoing FNA with concomitant liver biopsy and patients undergoing FNA alone. Patients were given analgesia in recovery upon request. The type of analgesia (opiate versus paracetamol) was also recorded. Adverse events and serious adverse events were collected in the immediate recovery period, as well as via phone call to the patient 1 week post-procedure. Further acceptability was assessed in the FNA alone group by determining the proportion of invited patients willing to take part in the study, as well as the proportion willing to undergo a second FNA (as assessed on questioning via telephone at 1 week post-FNA).

*Isolation of hepatic immune cells from liver biopsies and liver resection tissue*

Percutaneous liver biopsy was performed under ultrasound guidance with a Quick-core Biopsy needle set with a 2 cm stylet. Surgical liver resections were performed by hepatobiliary surgeons for removal of tumours with a sample taken from the edge of the explanted resection which represented non-tumour liver. In both cases, samples were placed in R10 media (RPMI-1640, 10% foetal calf serum (FCS), 1% penicillin/streptomycin, 1% L-glutamine) on ice. Liver biopsy samples weighed 20-30 mg each, and for liver resections sample portions of up to 80 mg were used for cell isolation.

Liver samples then underwent mechanical digestion and cell purification. Mechanical digestion was preferred to enzymatic digestion as per previously published protocols of lymphocyte isolation from human liver tissue, as enzymatic digestion with proteolytic enzymes has been shown to alter the expression of cell surface receptors (Lalor PF et al, 2010). In brief, the liver was dissected by scalpel into smaller pieces, passed through a 100micron mesh filter, washed in R10 culture media, and cells were isolated using a Percoll® 30%/70% gradient. The cells were washed again in R10 media, and were then used for flow cytometry analysis or were frozen at 80°C in freezing media (10% DMSO/90% FCS) for future analysis.

In a subset of resection samples, an FNA was taken directly from the liver resection (i.e. the needle placed onto the liver resection as opposed to onto a patient’s skin as detailed below). The FNA on the resection and handling of the specimen was performed as described below in section on FNA. This was to ascertain whether FNA from a liver resection explant differed from FNA obtained percutaneously. The resection from which the FNA was performed was then digested and processed as per above.

*Isolation of hepatic immune cells via FNA*

Ultrasound examination was used to identify a suitable position for the liver FNA, typically in the right mid-axillary line, 1-2 intercostal spaces above the costal margin. A 22 gauge spinal needle with an internal stylet was inserted to 2-3 cm into the liver parenchyma from the capsule. Cells were aspirated into a 10 mL syringe filled with 2 mL ice-cold sterile saline using repeated gentle back pressure 5-10 times. The needle was withdrawn 1-2 cm (without withdrawing from the body) then reinserted back into the liver, just under the capsule, but orientated in a slightly different direction when passing into the liver. Aspiration was repeated with a fresh syringe containing 2 mL ice-cold sterile saline, and then repeated again but without repositioning. Syringe contents were emptied through the spinal needle into 40 mL ice-cold media comprising RPMI-1640 with 1% L-glutamine, 10% FCS, 1% penicillin/streptomycin, 25 mM 4-(2-hydroxyethyl)-1-piperazineethanesulfonic acid (HEPES). The aspirate was passed through the spinal needle as this increased the liver lymphocyte yield.

The FNA sample was then centrifuged within 1 hour of obtainment at 447xg for 10 min at 4°C, and the pellet was resuspended in 1X PBS with 2% bovine serum albumin (BSA), supplemented with 25 mM HEPES and proceeded to either flow cytometry analysis or FACS. FNA samples were always analysed fresh (never frozen).

*Peripheral blood mononuclear cell (PBMC) isolation*PBMCs were isolated from fresh whole blood obtained by venesection by density gradient centrifugation (Lymphoprep) at 937x*g* for 30 min with no brake. The mononuclear layer was isolated and washed three times before being resuspended with 1X PBS with 2% BSA and 0.2% EDTA (or no EDTA if cells were used for downstream scRNA-seq).

*Flow cytometry*

Liver and peripheral blood mononuclear cells were transferred to a 96-well V-bottomed plate (up to 5x10^6^ cells/well), incubated for 10 min at 4°C with a blocking buffer (1:100 Human FcR blocker, Miltenyi, using 1 μL/1x10^6^ cells), and then washed with cell staining buffer. The cells were stained with antibodies (**Supplementary Table 1**) for 20 min at 4°C, and then washed. Samples incubated with 1X red blood cell lysis buffer (eBioscience) at room temperature for 10 min before being washed once. Subsequently, the cells were fixed with 4.2% formaldehyde at 4°C for 15 min, washed twice, resuspended in cell staining buffer, and kept at 4°C before acquisition on a flow cytometer (LSR II, BD Biosciences) within 72 h of staining. Acquired data were analysed using FlowJo^TM^ software version 10 (see **Supplementary Table 3** for a list of all software used in this manuscript).

*FACS*

After staining with anti-CD45 and a live-dead discriminating dye, cells were resuspended in 150 μL of 1X PBS and kept at 4°C. 1.5 mL sterile collection microtubes were pre-coated at 4°C overnight with sterile 10% BSA. Immediately prior to FACS, the 10% BSA was discarded, and the tubes were washed twice with 1X Dulbecco’s PBS, then filled with 10 μL of 1X Dulbecco’s PBS. Cells were sorted into these tubes on the BD FACS ARIA III, and gated on live, singlet, CD45^+^ and lymphoid cell populations.

*Sample processing for 10x Genomics single-cell 3’ library generation*

Sorted cells were prepared according to the manufacturer’s protocol using the 10x Genomics Chromium Single Cell 3’ v2 Reagent Kit. Only samples which had a minimum of 2,000 FACS-sorted cells were taken forward for scRNA-seq, and a maximum of 10,000 cells were loaded per channel of the chip.

*Single-cell RNA-seq preprocessing and quality control*

Single-cell transcriptomics data were processed using cellranger (v3.1.0) with the default human reference as supplied by 10x Genomics. The output count matrices for barcodes identified as cells (129,473 cells) were analysed using the Panpipes pipeline (https://github.com/DendrouLab/panpipes). Briefly, we retained the cells which satisfied the following criteria: <10% genes mapping to haemoglobin genes, <10% mitochondrial content, with detection of at least 200 genes. To exclude doublets we classified cells using scrublet (v0.2.1) and removed cells which had more than 2,500 genes and a maximum scrublet score 0.25. In total we retained 117,201 cells for downstream data analysis.

*Single-cell RNA-seq integration and clustering*

Log1p library-size normalised counts were used to estimate the top variable genes using Seurat v3. These were pruned of immunoglobulin genes and T-cell receptor genes to minimise non-informative cell clustering by T/B-cell clonotypes.

Cells were integrated using bbknn (v1.3.12), controlling for patient and tissue type as covariates. An explorative clustering was run on the integrated data to identify the major cell types (T/natural killer (NK) cells, B/plasmablast cells, and myeloid cells). These major cell types were then re-processed separately with the same batch correction procedure. Separate lineages were clustered using leiden algorithm. Custom markers and top differentially expressed genes (Wilcoxon test, false discovery rate (FDR)$<$0.05) were used to identify and annotate the clusters. Uniform manifold approximation and projection (UMAP) plots were calculated by building the K-nearest neighbour graph from the first 50 principal components and using k=30, and Euclidean distance and minimum distance=0.5. Upon inspection of UMAP and marker plots, clusters were merged into pseudobulks based on the similarity of their expression profile by expert manual curation.

*Single-cell RNA-seq differential abundance analysis*

For differential abundance analysis, cell cluster frequency was estimated for each cluster within its major cell type; only cell clusters with at least five cells in nine samples were included. A Wilcoxon matched-pairs signed rank test was employed with a Benjamini-Hochberg correction for multiple testing.

*Single-cell RNA-seq differential expression and cell-cell interaction analyses*

Differential expression was calculated for each pseudobulk using edgeR (v3.30.0), testing only clusters with more than 50 cells. Briefly, for each pseudobulk, we retained genes whose expression was at least three counts per million in at least three samples across the experimental conditions, the counts were normalised and dispersions were estimated across conditions. We fitted a negative binomial generalised linear model with quasi-likelihood test (glmQLFit). Genes with FDR<0.05 in each cluster and contrast were deemed differentially expressed. To identify differentially expressed genes co-occurring in known pathways we used Fisher test and gene set enrichment analysis (fgsea v1.14.0), and pathways (biocarta.v7.2, pid.v7.2, v7.2, kegg.v7.2, reactome.v7.2, c3.tft.v7.2) were downloaded from the MSigDB database (<http://www.gsea-msigdb.org/gsea/msigdb/collections.jsp>). Cell-cell ligand-receptor interactions were inferred using CellPhoneDB ([www.cellphonedb.org](http://www.cellphonedb.org)). The lower cutoff for expression proportion of any ligand or receptor in a cell type was set to 10%, and the number of permutations was set to 1,000.

*Single-cell RNA-seq data visualisation*

Parsing and plotting of the results of each analysis was done using Python (scanpy, seaborn v0.11.0, matplotlib 3.3.2) and R (ggplot2 within tidyverse v1.3.0, circlize 0.4.9, complexHeatmap v2.5.3).

*Statistical analyses*

Unless otherwise stated, unpaired or paired t-tests or one-way analysis of variance with Tukey’s multiple comparisons test were utilised for the comparison of normally distributed continuous variables. For non-normally distributed continuous variables, Mann Whitney or Kruskall-Wallis tests were employed for unpaired data, whilst Wilcoxon matched-pairs signed rank or Friedman tests with Dunn’s multiple comparisons were used for paired data. All other multiple testing comparisons were performed using the Benjamini-Hochberg method and where multiple testing was taken into account, adjusted *P*-values (*P_adj_*) are reported. Comparisons of categorical outcomes and proportions were undertaken using Fisher’s exact test or the Chi-square test. For all data other than the scRNA-seq data, all statistical analyses were carried out on GraphPad Prism Software v8.

**Reference:**

Lalor PF, Curbishley SM, Adams DH. *Identifying homing interactions in T-cell traffic in human disease.* Methods Mol Biol. 2010;616:231-52. doi: 10.1007/978-1-60761-461-6_15. PMID: 20379879.

**Supplementary Tables:**

**Supplementary Table 1. Antibodies used in this study**

| **Name** | **Citation** | **Supplier** | **Cat no.** | **Clone no.** |
| --- | --- | --- | --- | --- |
| Anti-CD3 BV785 | [1] | BioLegend | 317330 | OKT3 |
| Anti-CD4 BV480 | [2] | BD Biosciences | 566165 | SK3 |
| Anti-CD8 APC-R700 | [3] | BD Biosciences | 565192 | SK1 |
| Anti-CD45 FITC | [4] | BioLegend | 304005 | HI30 |
| Anti-CD45RA PE-Dazzle 594 | [5] | BioLegend | 304145 | HI100 |
| Anti-CD56 BV421 | [6] | BioLegend | 318327/8 | HCD56 |
| Anti-CD69 BV711 | [7] | BioLegend | 310943 | FN50 |
| Anti-CD161 PE-Vio770 | [8] | Miltenyi | 130-099-967/5 | 191B8 |
| Anti-β7 PE | [9] | BioLegend | 321203 | FIB504 |
| Anti-CCR9 APC | [10] | BioLegend | 358907 | L053E8 |
| Anti-TCR-V$a$7.2 BV605 | [11] | BioLegend | 351719 | 3C10 |
| Live/dead stain  Near-IR | [12] | Life Technologies | L10119 | - |

**Supplementary Table 2. Summary statistics of total, non-immune and immune cell yields and the percentage of immune cells by liver sampling method**

| **Cell yield/percentage** | **Summary metric** | **FNA (*n*=18)** | **Biopsy (*n*=5)** | **Resection (*n*=4)** |
| --- | --- | --- | --- | --- |
| Total live single cells | Median | 34,664 | 42,062 | 527,050 |
|  | 25^th^ percentile | 11,895 | 24,856 | 20,492 |
|  | 75^th^ percentile | 127,658 | 44,569 | 1,183,802 |
|  | *P*-value* | 0.7370 | | |
| Non-immune (CD45-) cells | Median | 2,436 | 2,123 | 37,768 |
|  | 25^th^ percentile | 1,342 | 658 | 2,056 |
|  | 75^th^ percentile | 49,608 | 37,090 | 305,342 |
|  | *P*-value* | 0.5469 | | |
| Immune (CD45+) cells | Median | 23,213 | 16,235 | 334,383 |
|  | 25^th^ percentile | 9,497 | 7,480 | 18,436 |
|  | 75^th^ percentile | 39,627 | 36,050 | 1,033,359 |
|  | *P*-value* | 0.2496 | | |
| % Immune cells out of total live single cells | Median | 86.17 | 93.62 | 90.35 |
|  | 25^th^ percentile | 50.52 | 17.54 | 69.02 |
|  | 75^th^ percentile | 95.36 | 98.03 | 93.63 |
|  | *P*-value* | 0.8271 | | |

*Kruskal-Wallis test

**Supplementary Table 3. Software and algorithms used in this study**

| **Software name** | **Manufacturer** | **Version** |
| --- | --- | --- |
| BBKNN | Polanski et al. 2020 [13] | - |
| Cell Ranger | 10x Genomics | 3.1.0 |
| CellPhone DB | https://www.cellphonedb.org/; Efremova et al. 2020 [14] | v2 |
| edgeR | Robinson et al. 2010 [15] | - |
| fgsea | Korotkevich et al. 2021 [16] | - |
| FlowJo^TM^ | https://www.flowjo.com/ | 10 |
| GraphPad Prism | https://www.graphpad.com/ | 8 |
| Python | https://www.python.org/ | v3.8.2 |
| R Studio and R environment | https//www.rstudio.com/ and https://cran.r-project.org/ | - |
| Scanpy | https://github.com/theislab/scanpy; Wolf et al. 2018 [17] | **-** |
| UMAP | https://arxiv.org/abs/1802.03426; McInnes et al. 2018 [18] | **-** |

­

**Supplementary Table 4. Baseline demographics of patients whose FNA samples were used for scRNA-seq**

| **Liver disease** | **Age (yr)** | **Sex** | **Brunt fibrosis stage*** | **LSM (kPa)** | **INR** | **Platelets (x10^9^/L)** | **ALP (IU/L)** | **ALT (IU/L)** | **Bilirubin (**$\boldsymbol{\mu}$**mol/L)** | **Albumin (g/L)** | **Hb (g/L)** |
| --- | --- | --- | --- | --- | --- | --- | --- | --- | --- | --- | --- |
| PSC-UC_1 | 54.2 | M | n/a | 12.3 | 1 | 378 | 552 | 162 | 55 | 30 | 126 |
| PSC-UC_2 | 39.5 | M | n/a | 18.1 | 1 | 314 | 352 | 70 | 38 | 35 | 140 |
| PSC-UC_3 | 45.4 | M | n/a | n/a | n/a | 313 | 382 | 134 | 23 | 44 | 143 |
| MASLD_1 | 52.0 | F | 4 | 17.1 | 1 | 200 | 88 | 42 | 15 | 40 | 134 |
| MASLD_2 | 26.7 | F | 3 | 11.4 | 1 | 353 | 67 | 52 | 13 | 36 | 134 |
| MASLD_3 | 34.3 | M | 2 | 5.4 | 1 | 240 | 127 | 118 | 8 | 45 | 156 |
| MASLD_4 | 53.2 | F | 1 | 4.6 | 0.9 | 302 | 87 | 27 | 7 | 41 | 140 |
| MASLD_5 | 37.0 | M | 1 | 9.0 | 0.9 | 221 | 49 | 100 | 8 | 42 | 141 |
| MASLD_6 | 57.5 | M | 3 | 15.4 | 1 | 196 | 81 | 62 | 10 | 42 | 172 |
| *Brunt fibrosis stage is a component of the Brunt scoring system for NAFLD. The Brunt fibrosis stage is marked from 1 to 4, where 1 represents minimal fibrosis, and 4 is advanced fibrosis, or cirrhosis. ALP, alkaline phosphatase; ALT, alanine transaminase; F, female; Hb, haemoglobin; INR, international normalised ratio; LSM, liver stiffness measurement; M, male; MASLD, metabolic dysfunction-associated steatotic liver disease; n/a; not applicable or available; PSC, primary sclerosing cholangitis; UC, ulcerative colitis. | | | | | | | | | | | |

**Supplementary Table 9. Deposited data**

| **Name of repository** | **Identifier** | **Link** |
| --- | --- | --- |
| Zenodo (Flow cytometry data) | *Will be available upon acceptance* | *Will be available upon acceptance* |
| European Genome-phenome Archive (EGA) (scRNA-seq data) | *Will be available upon acceptance* | *Will be available upon acceptance* |
| Chan Zuckerberg Initiative (CZI) Science CELLxGENE Data Portal (scRNA-seq data) | *Will be available upon acceptance* | *Will be available upon acceptance* |

**Supplementary Figure 1. Flow cytometry gating strategy**

Representative gating strategy for flow cytometric analysis of FNAs and matched PBMCs. FMO, fluorescence minus one; FSC, forward scatter; SSC, side scatter.

**
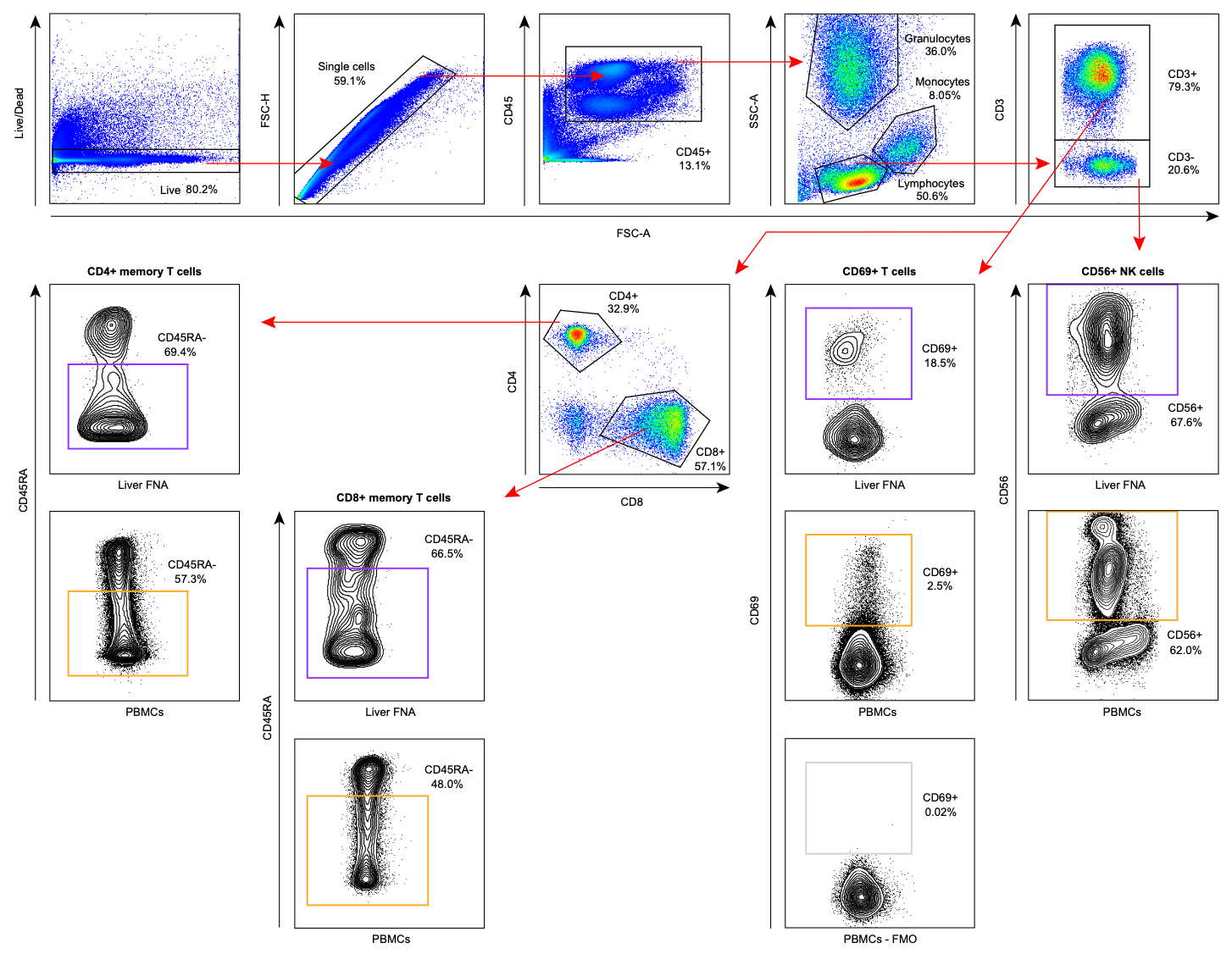
**

**Supplementary Figure 2. CD8^+^ T cell gating for MAITs, CCR9, and β7**

Representative liver FNA sample. Upper panel - CD8^+^ T cells gated on CD45RA, then CD8^+^ CD45RA^-^ T cells gated on CD161/Vα7.2 to reveal MAITs; lower panel – CD8^+^ CD45RA^-^ are gated on CCR9^+^ cells (left) and β7^+^ cells (right). MAIT, mucosal-associated invariant T cell.

**
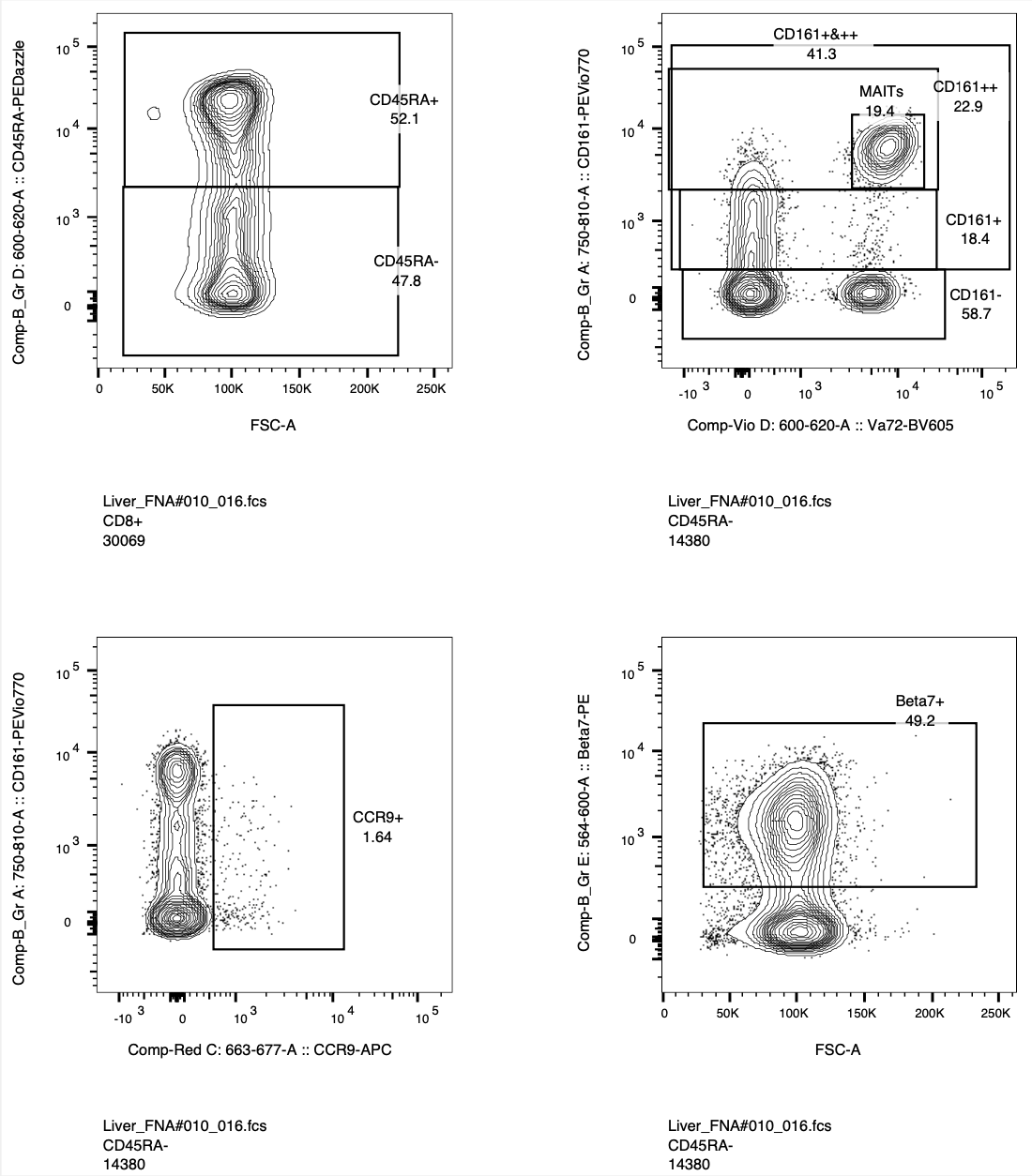
**

**Supplementary Figure 3. CD4^+^ T cell gating for CD161, CCR9, and β7**

Representative Liver FNA sample. Upper panel – CD4^+^ T cells gated on CD45RA, then CD4^+^ CD45RA^-^ T cells gated on CD161^+^ cells; lower panel – CD4^+^ CD45RA^-^ are gated on CCR9^+^ cells (left) and β7^+^ cells (right).

**Supplementary Figure 4. CCR9 and β7 expression on liver-derived T cells in PSC and controls**

Liver infiltrating lymphocytes were isolated from FNAs from patients with PSC and liver controls, who mainly comprised patients with MASLD (76% of patients as per Table 1 in the main manuscript). The proportion of CD4^+^ (left graphs) and CD8^+^ (right graphs) memory T cells which were CCR9^+^ (**A**) and β7^+^ (**B**) were compared. Bars depict the mean±SEM. Unpaired t-test or Mann Whitney test applied as appropriate. (**C**) There was no correlation between the proportion of CD4^+^ memory T cells which were CCR9^+^ and LSM. A line of best fit is shown, and Pearson correlation applied. FNA, fine needle aspirate; LSM, liver stiffness measurement; ns, not significant (*P*$\geq$0.05); PSC, primary sclerosing cholangitis.

**Supplementary Figure 5. CD161 and CD69 expression on liver-derived T cells in PSC and controls**

Liver infiltrating lymphocytes were isolated via FNA from patients with PSC and liver controls, who mainly comprised patients with MASLD (76% of patients as per Table 1 in the main manuscript). (**A**) The proportion of CD4^+^ memory T cells which were CD161^+^ (left) and the proportion of CD8^+^ memory T cells which were MAIT cells (CD161^++^ Vα7.2^+^, right) were compared between PSC and liver controls. There was a trend towards a higher proportion of MAITs in patients with PSC, which however was not statistically significant. (**B**) the proportion of CD4^+^ (left) and CD8^+^ (right) memory T cells which were CD69^+^ was compared between patients with PSC and liver controls. There was a higher proportion of CD8^+^ T cells expressing CD69 among patients with PSC compared with controls. Graphs depict mean±SEM; unpaired t-test applied. FNA, fine needle aspirate; MAIT, mucosal-associated invariant T cell; PSC, primary sclerosing cholangitis. *=*P*<0.05, ns=not significant (*P*$\geq$0.05).

**
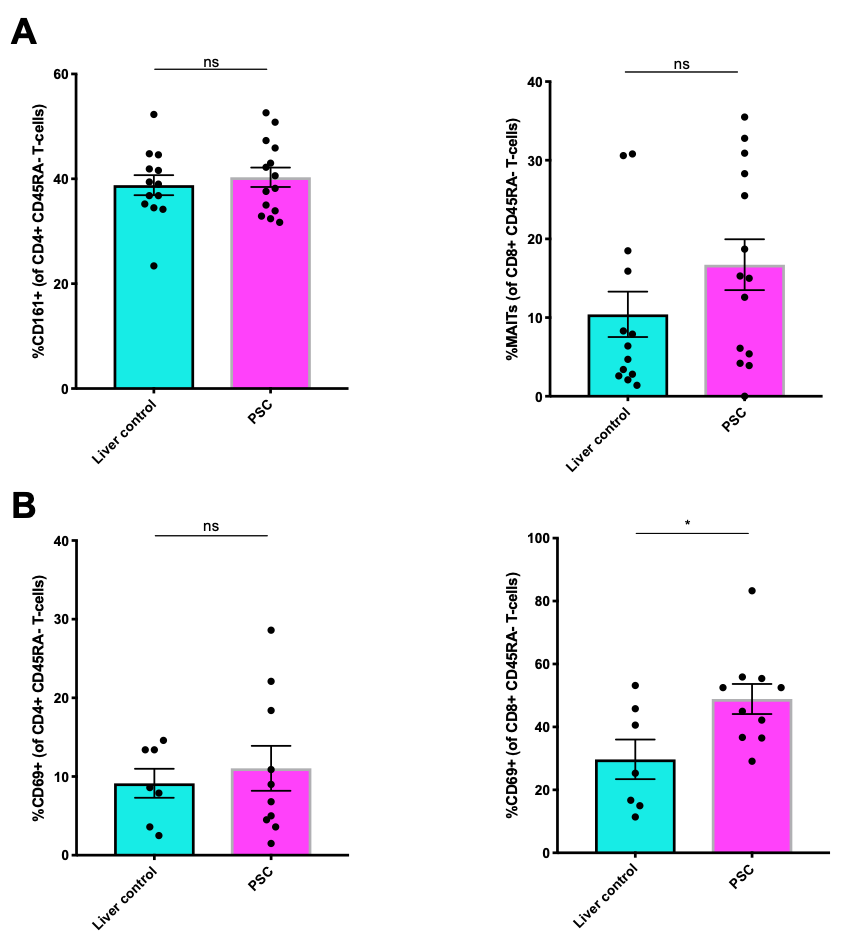
**

**References**

1. Lisovsky I, Isitman G, Song R, DaFonseca S, Tremblay-McLean A, Lebouche B, et al. A higher frequency of NKG2A+ than of NKG2A− NK cells responds to autologous HIV-infected CD4 cells irrespective of whether or not they coexpress KIR3DL1. J Virol 2015;89:9909-19.
2. Reichert T, DeBruyere M, Deneys V, Totterman T, Lydyard P, Yuksel F, et al. Lymphocyte subset reference ranges in adult Caucasians. Clin Immunol Immunopathol 1991;60:190-208.
3. Engleman EG, Benike CJ, Glickman E, Evans RL. Antibodies to membrane structures that distinguish suppressor/cytotoxic and helper T lymphocyte subpopulations block the mixed leukocyte reaction in man. J Exp Med 1981;154:193-8.
4. Oeztuerk-Winder F, Guinot A, Ochalek A, Ventura J-J. Regulation of human lung alveolar multipotent cells by a novel p38α MAPK/miR-17-92 axis. EMBO J 2012;31:3431-41.
5. Del Alcazar D, Wang Y, He C, Wendel BS, Del Rio-Estrada PM, Lin J, et al. Mapping the lineage relationship between CXCR5+ and CXCR5- CD4+ T cells in HIV-infected human lymph nodes. Cell Rep 2019;28:3047-60.
6. Vansaun MN, Mendonsa AM, Lee Gorden D. Hepatocellular proliferation correlates with inflammatory cell and cytokine changes in a murine model of nonalchoholic fatty liver disease. PLoS One 2013;8:e73054.
7. Sagebiel AF, Steinert F, Lunemann S, Korner C, Schreurs RRCE, Altfeld M, et al. Tissue-resident Eomes+ NK cells are the major innate lymphoid cell population in human infant intestine. Nat Commun 2019;10:975.
8. Poggi A, Zocchi MR, Carosio R, Ferrero E, Angelini DF, Galgani S, et al. Transendothelial migratory pathways of V delta 1+TCR gamma delta+ and V delta 2+TCR gamma delta+ T lymphocytes from healthy donors and multiple sclerosis patients: involvement of phosphatidylinositol 3 kinase and calcium calmodulin-dependent kinase II. J Immunol 2022;168:6071-7.
9. Sandborn WJ, Mattheakis LC, Modi NB, Pugatch D, Bressler B, Lee S, et al. PTG-100, an oral α4β7 antagonist peptide: preclinical development and phase 1 and 2a studies in ulcerative colitis. Gastroenterology. 2021;161(6):1853-64.
10. Yin S, Mao Y, Li X, Yue C, Zhou C, Huang L, et al. Hyperactivation and in situ recruitment of inflammatory Vδ2 T cells contributes to disease pathogenesis in systemic lupus erythematosus. Sci Rep 2015;5:14432
11. Balakrishnan A, Gloude N, Sasik R, Ball ED, Morris GP. Proinflammatory dual receptor T cells in chronic graft-versus-host disease. Biol Blood Marrow Transplant 2017;23:1852-60.
12. Wu T, Wieland A, Araki K, Davis CW, Ye L, Scott Hale J, et al. Temporal expression of microRNA cluster miR-17-92 regulates effector and memory CD8+ T-cell differentiation. Proc Natl Acad Sci USA 2012;109:9965-70.
13. Polanski K, Young MD, Miao Z, Meyer KB, Teichmann SA, Park J-E. BBKNN: fast batch alignment of single cell transcriptomes. Bioinformatics 2020;36:964-5.
14. Efremova M, Vento-Tormo M, Teichmann SA, Vento-Tormo R. CellPhoneDB: inferring cell–cell communication from combined expression of multi-subunit ligand–receptor complexes. Nat Protoc 2020;15:1484-506.
15. Robinson MD, McCarthy DJ, Smyth GK. edgeR: a Bioconductor package for differential expression analysis of digital gene expression data. Bioinformatics 2010;26:139-40.
16. Korotkevich G, Sukhov V, Budin N, Shpak B, Artyomov MN, Sergushichev A. Fast gene set enrichment analysis. bioRxiv 2021; https://doi.org/10.1101/060012.
17. Wolf FA, Angerer P, Theis FJ. SCANPY: large-scale single-cell gene expression data analysis. Genome Biol 2018;19:15.
18. McInnes L, Healy J, Melville J. UMAP: uniform manifold approximation and projection for dimension reduction. Arxiv 2018 arXiv:1802.03426.
